# Early detection of serum enteric bacterial DNA with real-time PCR in patients with SIRS

**DOI:** 10.1186/cc10400

**Published:** 2011-10-27

**Authors:** JM-C Yang

**Affiliations:** 1 Department of Surgery, Kaohsiung Veterans General Hospital, Taiwan, China

## Introduction

Sepsis remains a major and increasing healthcare problem with a mortality exceeding 25%. The early detection of infection is important in treating sepsis. Nucleic acid amplification methods have the potential to improve the timeliness, sensitivity, and accuracy of the tests used to detect respiratory pathogens. We used a quantitative real-time PCR (rt-PCR) to detect the enteric bacterial counts in blood from patients in the emergency room.

## Methods

EDTA samples were collected from patients with systemic inflammatory response syndrome (SIRS) presenting to the emergency room after obtaining informed consent. Enteric bacterial loads in blood samples were assayed by rt-PCR to quantitate the bacterial 23S rDNA and EB rDNA loads. Descriptive and clinical data were collected from the medical records and compared with 23S and EB rDNA results.

## Results

From January 2011 to April 2011, 39 patients (mean age 71.15 ± 17.12, range 22 to 93) were enrolled in the study. There was no correlation between serum lactate and enteric bacterial load in patients with SIRS. However, in a subgroup comprising patients presenting with respiratory distress and abnormal blood white cell count, the enteric bacterial rDNA load was higher and showed correlation with serum lactate level. The serum enteric bacterial rDNA loads were significantly higher in patients with positive cultures and in patients presenting with higher serum lactate. Correlations between serum lactate and enteric bacterial rDNA load were also significant in the patients with positive culture results.

## Conclusion

The quantitative assay for enteric bacterial rDNA could be a useful tool to detect early enteric bacterial translocation in patients presenting to the emergency room with elevated serum lactate level or with respiratory distress and abnormal white blood cell counts.

